# *In Vitro* Protective Effect and Antioxidant Mechanism of Resveratrol Induced by Dapsone Hydroxylamine in Human Cells

**DOI:** 10.1371/journal.pone.0134768

**Published:** 2015-08-18

**Authors:** Rosyana V. Albuquerque, Nívea S. Malcher, Lílian L. Amado, Michael D. Coleman, Danielle C. dos Santos, Rosivaldo Sa. Borges, Sebastião Aldo S. Valente, Vera C. Valente, Marta Chagas Monteiro

**Affiliations:** 1 Programa de Pós-graduação em Ciências Farmacêuticas, Faculdade de Farmácia, Universidade Federal do Pará/UFPA, Rua Augusto Corrêa, 01, Bairro Guamá, 66075–110, Belém, PA, Brasil; 2 Faculdade de Farmácia, Universidade Federal do Pará/UFPA, Belém, Pará, Brasil; 3 Instituto de Ciências Biológicas, Universidade Federal do Pará/UFPA, Belém, PA, Brasil; 4 Mechanisms of Drug Toxicity Group, School of Life and Health Sciences, Aston University, Aston Triangle, Birmingham, B4 7ET, United Kingdom; 5 Seção de Parasitologia, Instituto Evandro Chagas, SVS, MS, Pará, Brazil; Indian Institute of Toxicology Research, INDIA

## Abstract

Dapsone (DDS) hydroxylamine metabolites cause oxidative stress- linked adverse effects in patients, such as methemoglobin formation and DNA damage. This study evaluated the ameliorating effect of the antioxidant resveratrol (RSV) on DDS hydroxylamine (DDS-NHOH) mediated toxicity *in vitro* using human erythrocytes and lymphocytes. The antioxidant mechanism was also studied using *in-silico* methods. In addition, RSV provided intracellular protection by inhibiting DNA damage in human lymphocytes induced by DDS-NHOH. However, whilst pretreatment with RSV (10–1000 μM significantly attenuated DDS-NHOH-induced methemoglobinemia, but it was not only significantly less effective than methylene blue (MET), but also post-treatment with RSV did not reverse methemoglobin formation, contrarily to that observed with MET. DDS-NHOH inhibited catalase (CAT) activity and reactive oxygen species (ROS) generation, but did not alter superoxide dismutase (SOD) activity in erythrocytes. Pretreatment with RSV did not alter these antioxidant enzymes activities in erythrocytes treated with DDS-NHOH. Theoretical calculations using density functional theory methods showed that DDS-NHOH has a pro-oxidant effect, whereas RSV and MET have antioxidant effect on ROS. The effect on methemoglobinemia reversion for MET was significantly higher than that of RSV. These data suggest that the pretreatment with resveratrol may decrease heme-iron oxidation and DNA damage through reduction of ROS generated in cells during DDS therapy.

## Introduction

Leprosy, also known as Hansen’s disease, is a chronic infectious disease caused by the acid-fast bacillus *Mycobacterium leprae*. Primary transmission of the disease occurs from the extended exposure to oral or nasal secretions of ill and untreated patients, and by other transmission routes, such as contact with injured skin, blood, through vertical transmission, breast milk, insect bites [1], or by cutaneous inoculation with contaminated objects [2]. Leprosy remains a significant public health problem in 105 countries, as reported by the World Health Organization (WHO). Indeed, in 2012, 33,955 new cases were detected in Brazil alone [3] and epidemiological studies have reported that a high prevalence of leprosy remains in some regions of Brazil [4,5].

The current strategy for leprosy control recommended by the World Health Organization (WHO) is based on multidrug therapy (MDT) that consists of the combination of rifampicin, clofazimine and dapsone (4,4'-diaminodiphenylsulfone, DDS) for multi-bacillary leprosy patients and rifampicin and DDS for pauci-bacillary leprosy patients [3]. DDS therapy is responsible for hematological adverse reactions, such as methemoglobinemia and anemia [6,7]. These effects are associated with the hydroxylamine metabolite of DDS (DDS-NHOH) that is formed through N-hydroxylation by hepatic cytochromes P450, particularly CYP2C9 and CYP2C19 [6,8]. The principal targets of hydroxylamine compounds in humans are erythrocytes, involving mainly the production of high rates of methemoglobinemia and a significant reduction in erythrocytic lifespan [9]. In addition, some studies have reported cytogenetic damage and genotoxicity in leprosy patients under treatment with anti-leprotic drugs [10,11].

It has been demonstrated that antioxidant compounds can prevent methemoglobin formation induced by DDS-NHOH, such as ascorbic acid, curcumin, α-lipoic acid, and its dihydrolipoic acid reduced derivative dihydrolipoic acid (DHLA) [12,13]. In this regard, resveratrol (5–3,5,4'-trihydroxy-trans-stilbene, RSV) is a polyphenol commonly found in berries, grapes and peanut hulls, which exerts an significant antifungal action in these foods [14], as well as anti-inflammatory, antioxidant and antitumor actions in mammalian systems [15,16]. This natural non-flavonoid polyphenol has an important role in the prevention of human diseases such as cancer, cardiovascular diseases, diabetes and Alzheimer’s disease due its antioxidant properties [16,17,18].

In the present study, we aimed to evaluate *in vitro* effects of DDS-NHOH on methemoglobinemia induction, DNA damage and oxidative stress parameters, such as reactive oxygen species formation and antioxidant enzymes activity, as well as the protective effect of RSV on these alterations. The antioxidant mechanisms seen in the biological results, such as electron transfer processes were also explored using *in silico* molecular modeling techniques.

## Materials and Methods

### Chemicals

RSV, 2’,7’-Dichlorodihydrofluorescein diacetate (DCFH-DA), tert-butylhydroperoxide (t-BHP), methanol, ethanol, dimethyl sulfoxide, Triton X-100, sodium hydroxide, sodium chloride, ethylenediamine tetraacetic acid (EDTA) agarose for routine, agarose low electroendoosmosis (EEO), hydrogen peroxide (H_2_O_2_), hypoxanthine, ethidium bromide, methylene blue (MET), xanthine oxidase and cytochrome C were purchased from Sigma Chemical Com. (St. Lois, MO, USA). DDS hydroxylamine was purchased from Santa Cruz Biotechnology (Santa Cruz, CA). phytohemagglutinin M. was purchased from Life Technologies (Carlsbad, CA).

### Preparation of RSV and DDS-NHOH solutions

Resveratrol was dissolved in 100% ethanol as a stock of 0.2 mol/L and stored at -20°C and diluted in needed concentrations (10, 100, 200 and 1000 μM) with PBS 0.5 M, pH 7.4, before use. The final concentration of ethanol in the PBS was less than 0.001%. DDS-NHOH was dissolved in methanol and stored at -20°C.

### Ethics statement

This study was approved by The Ethical Committee of the Federal University of Pará, Brazil (protocol 165/11 CEP-ICS/UFPA) and informed consent was obtained from all subjects prior to the sample collection and experiment commencement. Thus, all participants were informed about the aims and methods of study and they wrote and signed the informed consent before the start of the experiment and sample collection. Non-smoking and non-drinking human venous blood from different healthy volunteers (both sexes and ages of 20–45 years) was obtained by venipuncture in heparin (5000 IU/ml) after an overnight fast.

### Preparation of erythrocyte suspensions

The blood was centrifuged at 3000 rpm for 10 min at 4°C. After removal of plasma, the buffy coat were removed and the isolated erythrocytes were washed three times with cold phosphate buffered saline (PBS; 0.9% NaCl, 10 mM Na_2_HPO4, pH 7.4) and the packed red blood cells (RBC) obtained were suspended (at 40% haematocrit) in the same solution.

### Pretreatment of erythrocytes with RSV or MET and treatment with DDS-NHOH

The methemoglobin formation and oxidative stress generation were induced *in vitro* by incubating erythrocytes suspension (40% haematocrit) with DDS-NHOH (2.5, 5.0, 7.5 and 10.0 μg/ml) or vehicle for 60 min at 37°C, as described by McMillan et al., [19]. The protective effect of RSV was evaluated by pre-incubating of erythrocytes suspension with this compound (10, 100, 200 or 1000 μM) for 60 min at 37°C [20]. Erythrocytes were then exposed to DDS-NHOH (2.5, 5.0, 7.5 and 10.0 μg/ml) for additional for 60 min at 37°C [19]. In addition, to assess effect of MET, the erythrocytes suspension was pre-incubated for 30 minutes with this substance (40 nM) and it was exposed to DDS-NHOH [21]. Cellular viability was analyzed prior and after the incubations.

### Post-treatment of erythrocytes with RSV or MET and treatment with DDS-NHOH

The reduction of methemoglobin formation of RSV was evaluated was evaluated as follows: Erythrocytes were exposed to DDS-NHOH (2.5 μg/ml) for 60 min at 37°C [19], after these cells were post-treated with 100 μM of RSV for 60 min at 37°C [20]. In addition, to assess effect of MET, the erythrocytes suspension was exposed to DDS-NHOH and after they were post-incubated for 30 minutes with MET (40 nM), as described by Reilly et al. [21]. Cellular viability was analyzed prior and after the incubations.

### Determination of Methemoglobin Content

Methemoglobin was determined according Hegesh et al. [22]. Methemoglobin content was evaluated in the buffered hemolysate through potassium cyanide-mediated conversion to cyanomethemoglobin, which absorb at a wavelength of 632 nm. A dilution of the hemolysate, in which potassium ferricyanide (K_3_Fe(CN)_6_) was used to convert all possible forms of hemoglobin (Hb) to methemoglobin, was used as a reference solution. The methemoglobin content was measured in duplicate, and values less than 2% were considered normal.

### Cell Culture and Sample Preparation to comet assay

Human venous blood was removed from several healthy volunteers who were abstainers from alcohol and tobacco (both sexes and ages of 20–45 years). Blood was obtained by venipuncture in heparin (5000 IU/ml). Briefly, 0.3 mL of venous blood were added to 4 mL of RPMI-1640 medium containing 20% of bovine calf serum and 50 μg/mL phytohemagglutinin. The mixture was then incubated at 37°C in a 5% CO2 incubator for 24 h, as previously described by Cao et al. [23]. Thus, lymphocytes were harvested with different drugs, as described below, and each sample were tested for viability using the trypan blue dry exclusion technique, as described by Cao et al. [23]. Only cell samples whose viability was over 90%, were measured by the Comet assay.

### DNA damage using Comet assay

Lymphocytes were treated with DDS-NHOH (7.5 μg/mL) and/or RSV (100 μM) for 3 hrs. After the cell viability determination (90%), comet assay was performed as described by Anderson et al. [24]. To perform the Comet assay, each sample was mixed with low melting-point agarose at 37°C, to a final concentration of 0.5%. The mixture (100 μL) was pipetted onto slides pretreated with 1.5% normal-melting-point agarose, to retain the agarose cell suspension. The drop containing the cells was covered with a glass cover slip (24 mm × 24 mm) and left at 4°C for 5 min. The cover slips were gently removed and the slides were then ready for processing. The slides were treated with a lysis solution (2.5 M NaCl, 100 mM EDTA, 100 mM TRIS, 1% Triton X-100, 10% DMSO, pH ~ 10,2) for 24h at 4°C. After protein removal, the slides were placed horizontally on an electrophoresis tray and the resultant nucleoids were immersed in electrophoresis buffer (300 mM NaOH, 100 mM EDTA, pH > 13) for 20 min at 4°C to cleave the alkali-labile sites. Then the electrophoresis was started using an electric field of 23 V/cm for 20 min. At the end of the process, the slides were gently removed from the tray and washed with distilled water for 5 min for neutralization.

The slides were dehydrated when immersed for 3 min in absolute ethanol and were then air dried. Finally, the slides were stained with ethidium bromide (20 μg/mL) and viewed using fluorescence microscopy ZEISS AxioCam HRc with green barrier filter 510–560 nm and 400 x coupled to a video camera. The cell images were analyzed using Tritek Comet Score Freeware 1.6 software. Registered parameters included the percent of DNA in the tail (Tail DNA %), Tail Length (TL), Tail Moment (TM) and Olive Moment (OM) as marker of DNA damage. One hundred comets were scored randomly for each concentration employed. All experiments were performed in duplicate and hydrogen peroxide (H_2_O_2_; 200 μM) was employed as a positive control, which caused pronounced DNA damage and confirmed the accessibility of the cells to the tested chemicals. All steps described previously were carried out in a darkroom, to prevent the interference of additional DNA damage.

### Measurement of intracellular reactive oxygen species (ROS)

ROS production induced by DDS-NHOH (2.5 and 7.5 μg/ml) in human erythrocytes was evaluated using 2’, 7’-Dichlorodihydrofluorescein diacetate (DCFH-DA). Erythrocytes suspensions were pretreated with RSV (100 and 1000 μM) for 1 h at 37°C and subsequently these cells were exposed to DDS-NHOH or T-BHP for 30 min [25]. The t-BHP (200 μM), an organic peroxide widely used in a variety of oxidation processes, was used as positive control [26]. Twenty minutes before the end the exposure with DDS-NHOH, 10 μM DCFH-DA was added to the suspension and incubated for 30 minutes at 37°C. Immediately, the DCF fluorescence intensity was measured by flow cytometry (FACSCanto, Becton Dickinson LSR II flow cytometer, San Jose, CA, USA) at an excitation wavelength of 488 nm and a 530 nm emission filter [27, 28]. When applied to intact cells, the nonionic, nonpolar, non-fluorescent DCFH-DA crosses cell membranes and is hydrolyzed enzymatically by intracellular esterases to form the intermediate to non-fluorescent 2,7-dichlorodihydrofluorescein (DCFH) that reacts with various ROS (including H_2_O_2_, OH^•^, and O_2_^•−^) and also by RNS (^•^NO and ONOO^-^) to form 2’,7’-dichlorofluorescein (DCF), a highly fluorescent product [29]. Thus, some authors considered the DCFH as a sensible probe, that not only measures the H_2_O_2_ in presence of cellular peroxidases, but also determinate the ONOO^¯^, and HO^•^ [29, 30].

### Superoxide Dismutase (SOD) Activity

Determination of SOD activity was performed according to the procedure recommended by McCord and Fridowich [31]. This method evaluated the ability of SOD to catalyze the conversion of O_2_^-^ to H_2_O_2_ and O_2_. SOD activity was measured using UV spectrophotometry at a wavelength of 550 nm and was expressed in nmol/mL. The T-BHP (200 μM) also was used as positive control.

### Catalase Activity

Catalase (CAT) activity was determined following the method described by Aebi [32], measuring the rate of enzymatic decomposition of H_2_O_2_ (10 mM) at 240 nm. Enzyme activity was expressed in CAT units, where one unit is the amount of enzyme needed to hydrolyze one μmole of H_2_O_2_/min/mg protein, at 37°C and pH 8.0.

### Data analysis

Data are reported as the mean ± S.E.M values. Data of methemoglobin formation was analyzed by employing Mann-Whitney non-parametric test. For the other parameters, statistically significant differences between groups were determined using Analysis of Variance (ANOVA) followed by the Tukey multiple comparison tests. In all cases, the significance level adopted was 5% (α = 0.05).

### Molecular Modeling

The theoretical calculations were carried out with the GAUSSIAN 03W program [33], within the density functional theory (DFT) approach, using the B3LYP functional, which includes a mixture of Hartree–Fock (HF) and DFT exchange terms. The gradient-corrected correlation functional [34,35] was used parameterized after Becke [36,37], along with the double-zeta split valence basis sets 6-31G* [38]. Molecular geometries were fully optimized by the Berny Algorithm, using redundant internal coordinates [39]. In order to study the barriers of internal rotation, the geometries were optimized with different dihedral angles. Since our interest is to understand the role played by the possible action mechanism of these molecules, we adopted a systematic study comparing electronic properties of DDS-NHOH with MET and RSV. The better correlation was obtained using the lowest ionization potential and stabilization energies in electron volts (eV). In order to achieve this aim, we calculated the following properties: (i) Highest Occupied Molecular Orbital (HOMO); (ii) Ionization Potential (IP); (iii) stabilization energy (ΔEiso). The IP was calculated as the energy difference between a neutral molecule and the respective cation free radical (Eq 1). The ΔEiso was determined by the calculation of one-electron redox energy between two molecules (1 and 2), as showed in Eq 2.

$$\text{IP}=E{\text{M}}^{•+}-E\text{M}$$ (1)

$$△\text{Eiso}=(E{\text{M}1}^{•+}+E\text{M}2)-(E\text{M}1+E{\text{M}2}^{•+})$$ (2)

## Results

### MetHb formation induced by DDS-NHOH

**Fig 1:**
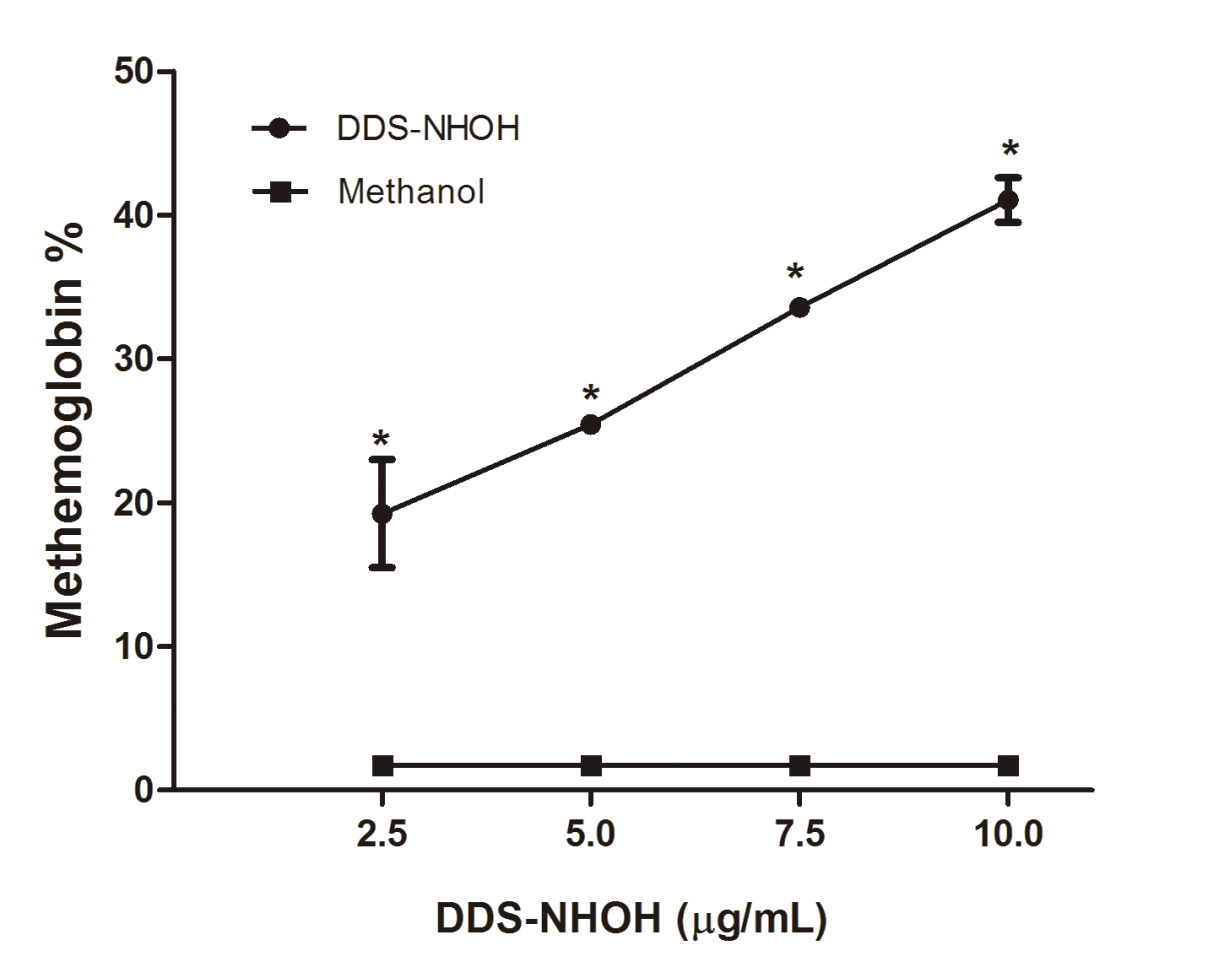
Effect of the DDS-NHOH on methemoglobin formation in human erythrocytes. Erythrocytes were incubated with different concentrations of DDS-NHOH (2.5; 5.0 and 7.5 μg/mL) for 1 h at 37°C. Data are reported as means ± S.E.M from three independent experiments done in triplicate. *P < 0.05 compared to methanol group.

The highest DDS-NHOH concentration induced the greatest methemoglobin formation, showing a dose-dependent effect. The levels of methemoglobin in the negative control (methanol) were less than 2%, corresponding to values within the normal range (Fig 1, S1 Table).

### Effect of RSV on methemoglobin formation induced by DDS-NHOH

**Fig 2:**
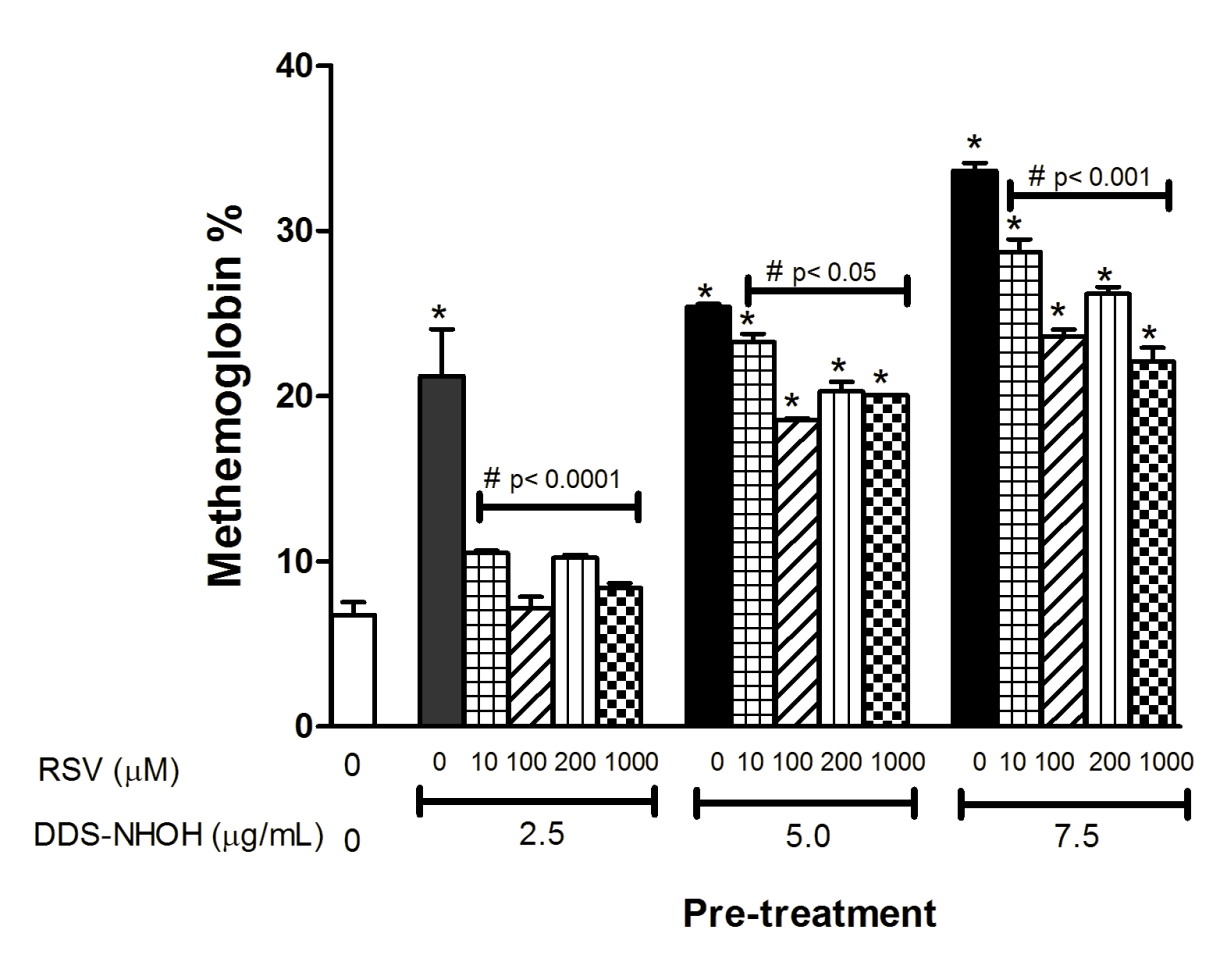
Effect of the pretreatment with different concentration of resveratrol (RSV) on methemoglobin formation induced by DDS-NHOH. Erythrocytes were pretreated with different concentrations of RSV(10, 100, 200 and 1000 μM) for 1 h at 37°C, then these cells were incubated with different concentrations of DDS-NHOH (2.5; 5.0 and 7.5 μg/mL) for 1 h at 37°C. Data are reported as means ± S.E.M from three independent experiments done in triplicate. ^**#**^P < 0.05 compared to DDS-NHOH group.

To evaluate the effect of RSV in methemoglobin formation induced by DDS-NHOH (2.5; 5.0 and 7.5 μg/ml), erythrocytes were pretreated with different concentrations of RSV (10; 100; 200 and 1000 μM) for 60 min and, after these cells were incubated with DDS-NHOH (2.5; 5.0 and 7.5 μg/ml). Pre-treatment with RSV inhibited the methemoglobin formation induced by DDS-NHOH at all concentrations. The best protective effect of RSV (in all tested concentrations) on methemoglobin formation was observed at 2.5 μg/ml of DDS-NHOH. Of the concentrations of RSV used in this study, 100 μM of the compound attenuated methemoglobin formation in a similar manner to 200 to 1000 μM concentrations (Fig 2, S2 Table). Therefore, 100 μM RSV was adopted for subsequent experiments.

### Comparative Effect of pretreatment with RSV or MET on methemoglobin formation induced by DDS-NHOH

**Fig 3:**
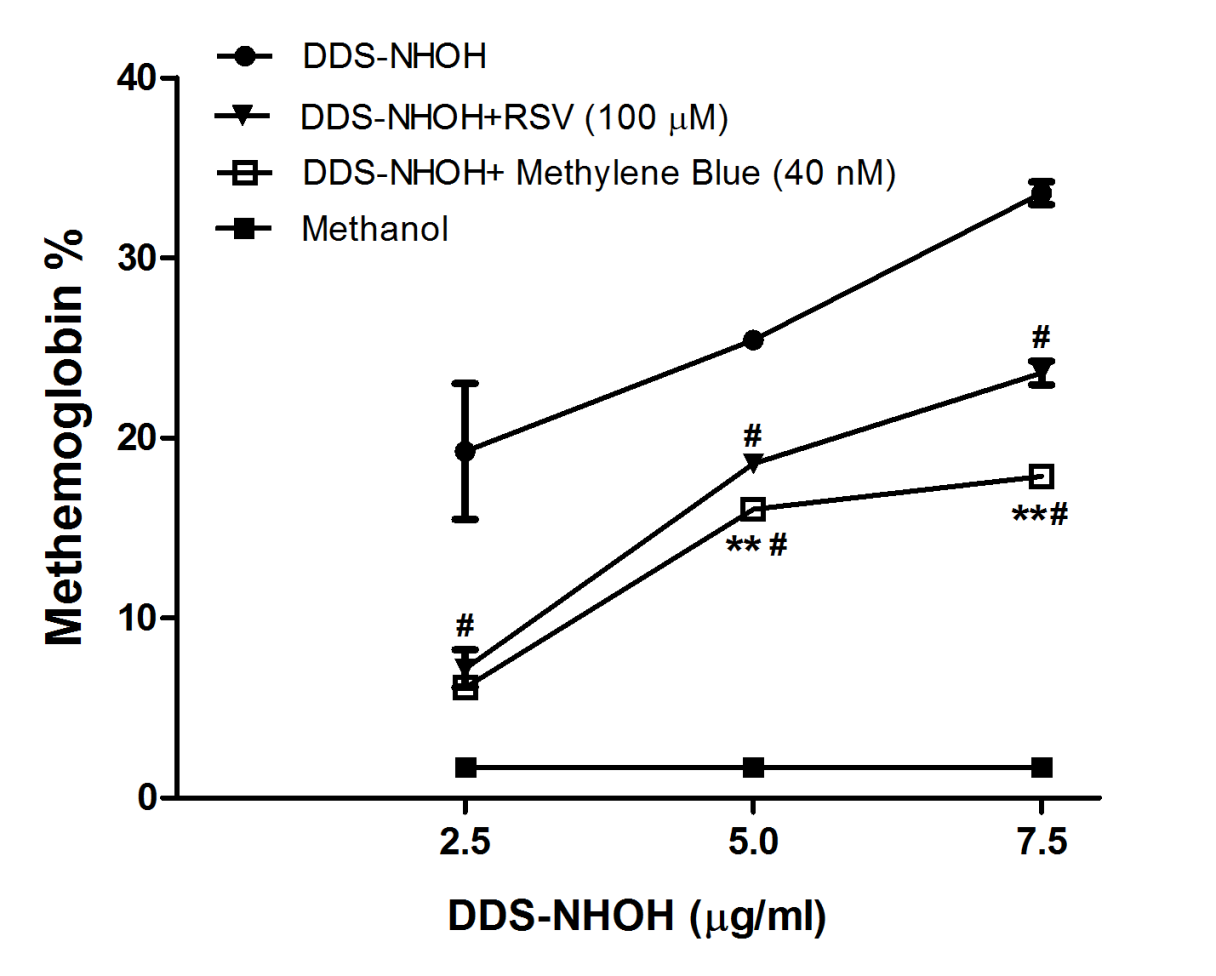
Comparative effect of the pretreatment with resveratrol (RSV) or methylene blue (MET) on methemoglobin formation induced by DDS-NHOH. Erythrocytes were pre-incubated with RSV (100 μM) for 1 h or MET (40 nM) for 30 min, after these cells were incubated for 1 h with different concentrations of DDS-NHOH (2.5, 5.0 and 7,5 μg/mL). Data are reported as mean ± S.E.M. ^**#**^P < 0.05 compared to DDS-NHOH group.**P < 0.05 compared to resveratrol group.

MET is a standard anti-methemoglobinemic treatment which is used in cases of DDS intoxication, so we evaluated the pretreatment effect of RSV and MET on methemoglobin formation. Erythrocytes were pretreated with MET (40 nM) or RSV (100 μM) and later these cells were incubated with DDS-NHOH (2.5, 5.0 and 7.5 μg/ml). Our data showed that the MET, even at a concentration of at least 2000-fold lower than the RSV, was significantly more effective in inhibiting the methemoglobin formation induced by all DDS-NHOH concentrations evaluated than RSV; this was most marked at concentrations of 5.0 and 7.5 μg/ml of DDS-NHOH (Fig 3, S3 Table).

### Comparative Effect of post-treatment with RSV and MET on methemoglobin formation induced by DDS-NHOH

**Fig 4:**
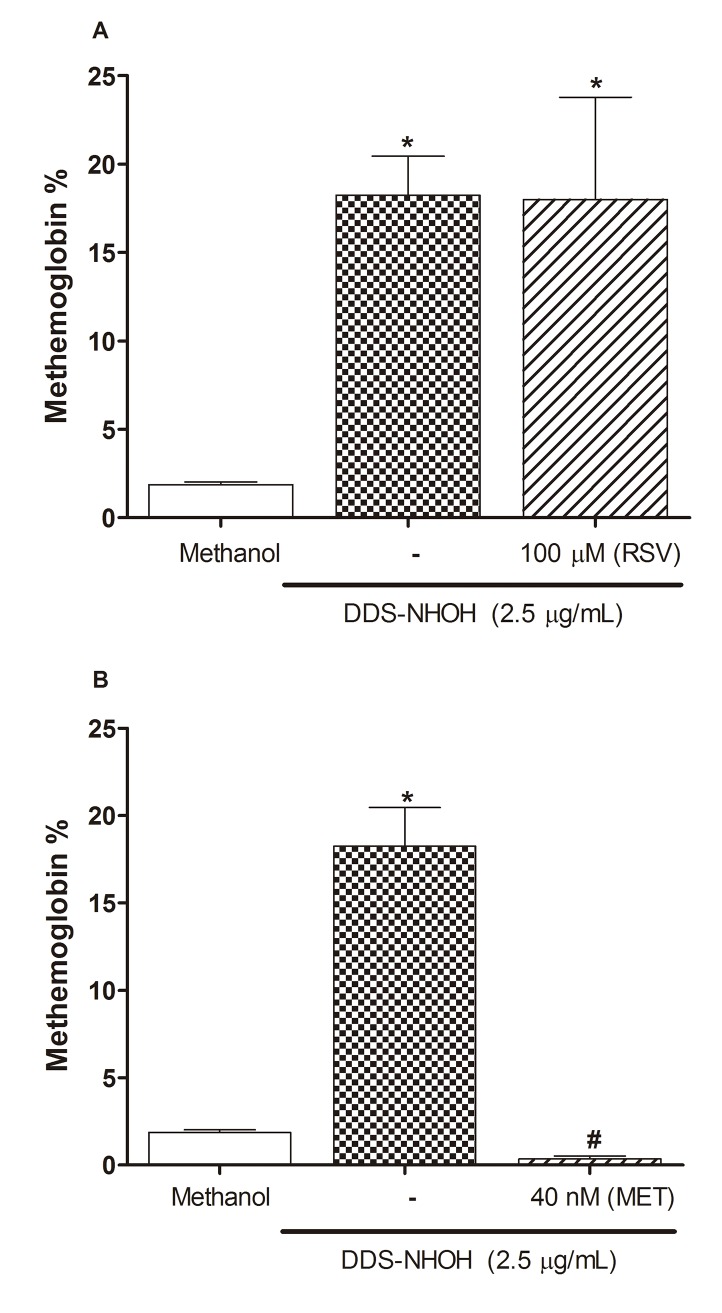
Comparative effect of post-treatment with resveratrol (RSV) or methylene blue (MET) on methemoglobin formation induced by DDS-NHOH. Erythrocytes were incubated for 1 h with DDS-NHOH (2.5 μg/mL), then these cells were incubated with RSV (100μM) for 1 h or MET(40 nM). Data are reported as mean ± S.E.M. *P < 0.05 compared to methanol group. ^#^P < 0.05 compared to DDS-NHOH group.

We also evaluated the post-treatment effect of RSV and MET on methemoglobin formation. For this, erythrocytes were incubated with DDS-NHOH (2.5 μg/ml) and after cells were post-treated with MET (40 nM) or RSV (100 μM). Data showed that the RSV did not reverse the methemoglobin formation induced by DDS-NHOH (Fig 4A, S4 Table), however, the MET fully reversed this methemoglobin formation, returning the methemoglobin values to those of the methanol control group (Fig 4B, S4 Table), demonstrating the superiority of MET in comparison with RSV in DDS-NHOH-induced methemoglobin reversal.

### Effect of RSV on DNA damage induced by DDS-NHOH

**Fig 5:**
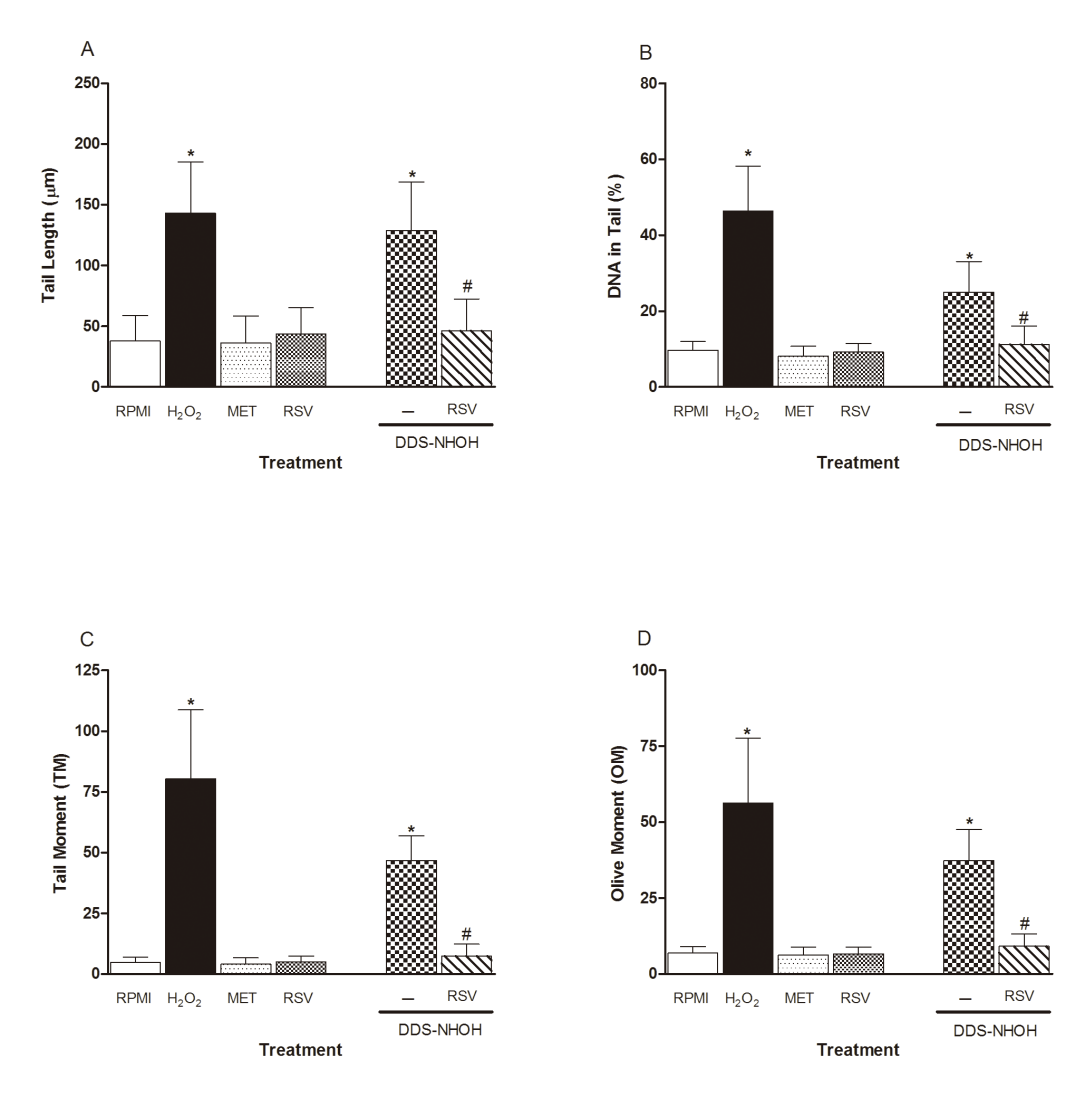
Effect of treatment with resveratrol on DNA damage induced by DDS-NHOH. Tail Length (μm—**A**), DNA in tail (%—**B**) Tail Moment (TM—**C**) and Olive Moment (OM—**D**) were used as a marker of DNA damage in lymphocyte using Comet assay. As positive control was used H_2_O_2_ (200 μM). All values are depicted as mean ± S.E.M.

In order to assess the effect of RSV in DNA damage induced by DDS-NHOH, peripheral blood lymphocytes were pretreated with RSV (100 μM) and later these cells were incubated DDS-NHOH (7.5 μg/ml) and DNA damage was assessed using the Comet assay. Fig 5A–5D) and S5 Table showed that the DDS-NHOH induced higher DNA fragmentation (% DNA in Comet tail, Tail Length (%), TM and OM) compared with vehicle control (methanol), but with values similar to positive control (H_2_O_2_). Moreover, RSV was able significantly to attenuate DNA damage induced by DDS-NHOH, to undetectable levels.

### The effect of RSV on DDS-NHOH-induced oxidative stress

#### Flow cytometric measurement of ROS production with DCFH-DA

**Fig 6:**
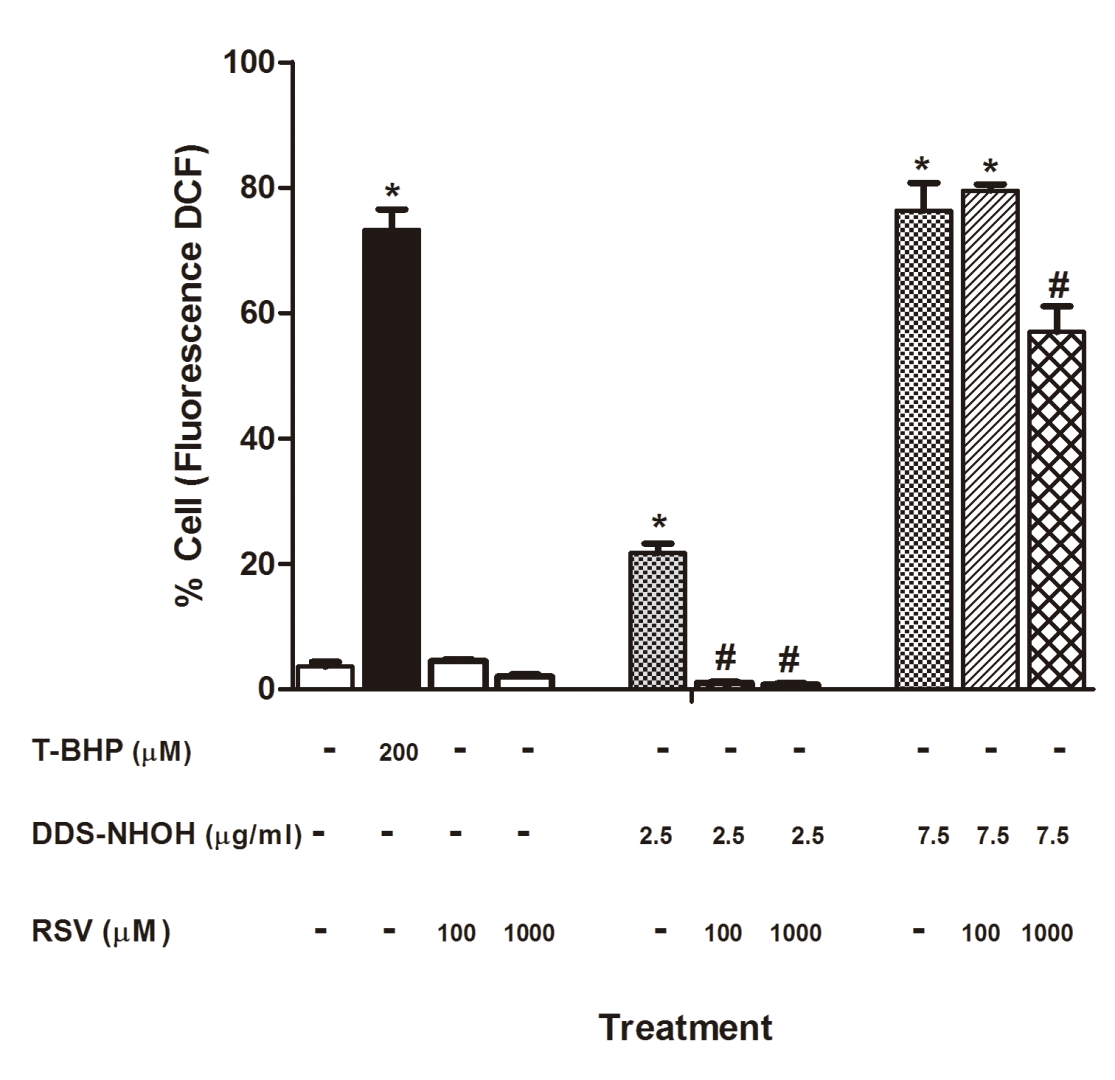
Reactive oxygen species (ROS) generation. Erythrocytes were pretreated with resveratrol (RSV, 100 μM and 1000 μM) for 1 h at 37°C and incubated for 30 min with DDS-NHOH (2.5 μg/ml and 7.5 μg/ml). As positive control was used T-BHP (200 μM). ROS production was measured as dichlorofluorescein (DCF) fluorescence. Values are means ± S.E.M. *P < 0.05 compared to methanol group. **#**P < 0.05 compared to DDS-NHOH group.

DDS-NHOH (7.5 μg/ml) and T-BHP (positive control) induced higher levels of intracellular ROS than other DDS-NHOH concentrations, but ROS formation was also detected in of 2.5 μg/ml DDS-NHOH exposed erythrocytes. Regarding pretreatment with RSV, all concentrations (100 and 1000 μM) were able to inhibit ROS production induced by DDS-NHOH (2.5 μg/ml), although only the concentration of 1000 μM of RSV reduced the ROS production induced by DDS-NHOH (7.5 μg/ml) (Fig 6, S6 Table).

#### Effects of DDS-NHOH on CAT and SOD activities and effect of the pretreatment with RSV

**Fig 7:**
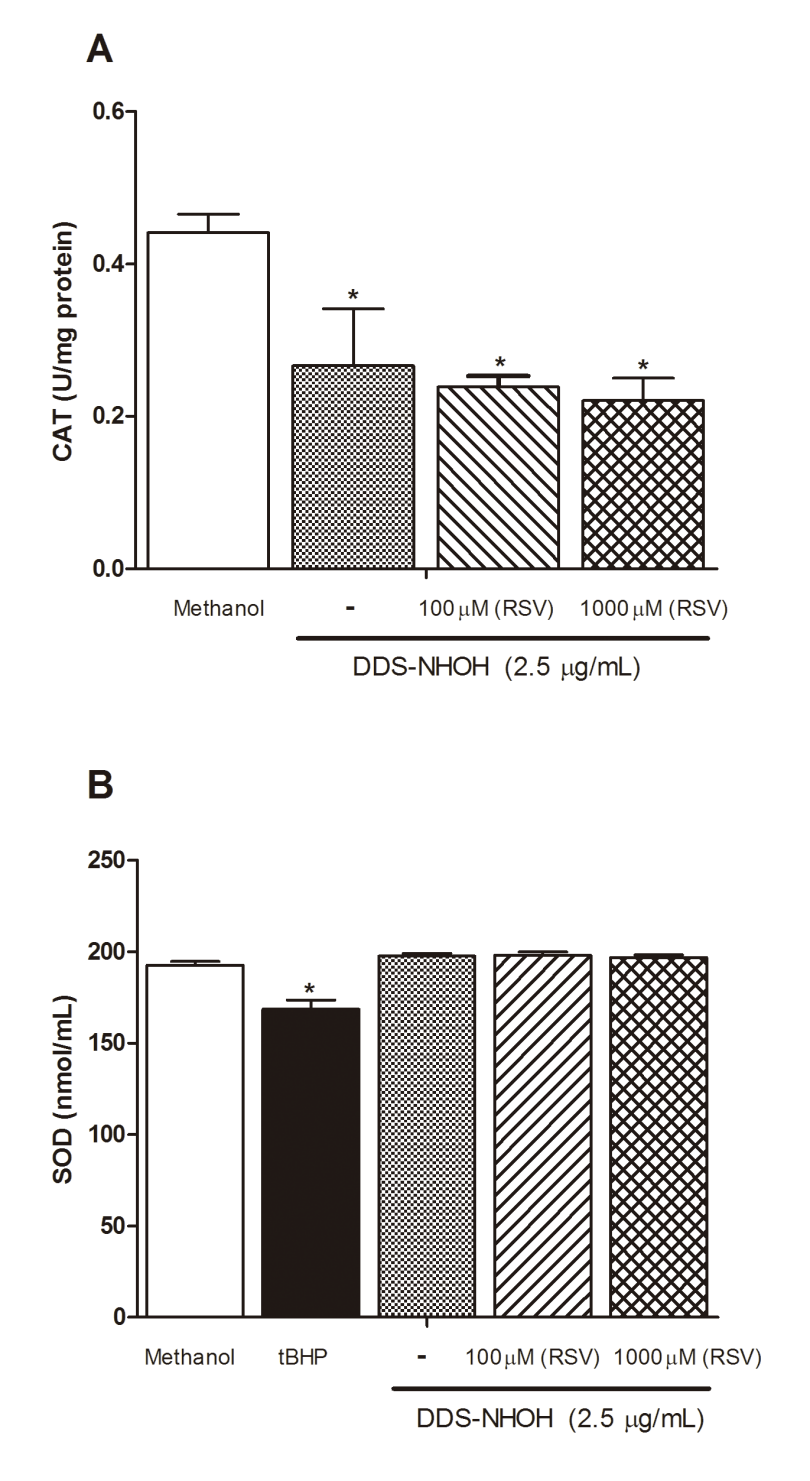
CAT and SOD activity. Erythrocytes were pretreated with resveratrol (RSV, 100 μM and 1000 μM) for 1 h at 37°C and incubated for 30 min with DDS-NHOH (2.5 μg/ml) or T-BHP (200 μM). Results are expressed as mean ± S.E.M. *P < 0.05 compared to methanol group.

DDS-NHOH significantly reduced CAT activity when compared to negative control, but did not alter the activity of SOD. The t-BHP (200 μM) was able significantly to reduce SOD activity when compared to the negative control. However, concentrations of RSV did not alter CAT and SOD activities in erythrocytes incubated with DDS-NHOH (Fig 7A and 7B and S7 Table).

### Theoretical Antioxidant Mechanism

**Fig 8:**
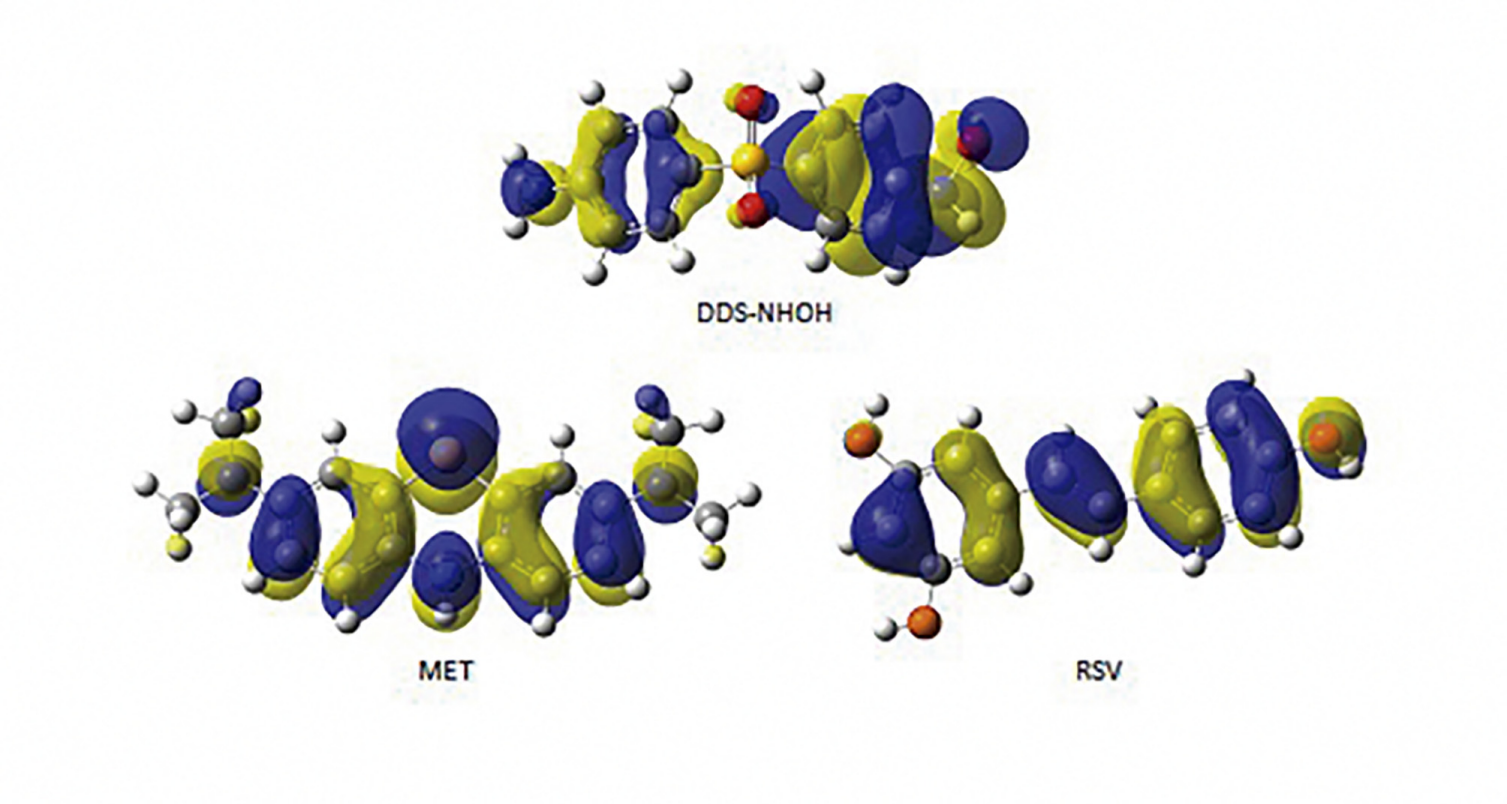
HOMO surface. Structure for HOMO of the dapsone hydroxylamine (DDS-NHOH), resveratrol (RSV), and methylene blue (MET). All nodal patterns related to individual group contributions are presented by blue or yellow for negative or positive wave function, respectively.

Molecular orbital calculations provided a detailed description of the orbitals including the spatial characteristics, nodal patterns, and individual atom contributions. The contour plots of the frontier orbitals for the ground state of DDS-NHOH, MET, and RSV are shown in Fig 8, where we can see mainly the highest occupied molecular orbital (HOMO) related to their nucleophilic properties. All nodal patterns related to individual group contributions are presented by blue or yellow for negative or positive wave function, respectively.

It was interesting that these orbitals were substantially distributed over the conjugation plane. In fact, all molecular compounds that presented a π-type electron system, where the resonance effects or canonical forms were located predominantly on heterocyclic, aniline or phenol rings. Thus, the substitution influenced the electron donation ability while imposing a high impact on the electron donating ability. Therefore, the more contributions are related to high nucleophilic regions. In addition, the orbital energy levels of the HOMO and LUMO of compounds studied here are listed in Table 1. All values are given in eV.

**Table 1:** Theoretical parameters for redox mechanism. HOMO, LUMO, GAP, Ionization potential (IP) and stabilization energy (ΔEiso) of DDS hydroxylamine (DDS-NHOH), resveratrol (RSV), and methylene blue (MET). All values are given in eV.

Compound	HOMO	LUMO	GAP	IP	ΔEiso
DDS-NHOH	-6.12	-1.24	4.87	11.69	0
MET	-4.06	-0.28	3.77	5.43	-2.10
RSV	-5.58	-0.62	3.95	7.06	-0.50

An electronic system with a larger HOMO-LUMO gap should be less reactive than one having a smaller gap. In the present study, the HOMO-LUMO gap values of DDS-NHOH, MET, and RSV by DFT/B3LYP-6-31+G (d,p) methods were 4.87, 3.77, and 3.95 eV, respectively. The lower value in the HOMO and LUMO energy gap would explain the eventual charge-transfer interaction-taking place within the molecules. The low HOMO value for MET indicated that this molecule had low ionization energies, suggesting that it could lose electrons easily. These results indicated that MET was potentially better antioxidant when compared with RSV. However, the higher gap value for DDS-NHOH has indicated that it has a low antioxidant property.

These results can be explained due to the number of electron-donating groups. In fact, MET have more electron donating groups than RSV. Moreover, DDS-NHOH has an electron-withdrawing group between aniline and hydroxyl-aniline group, clarifying its low antioxidant potential.

**Fig 9:**
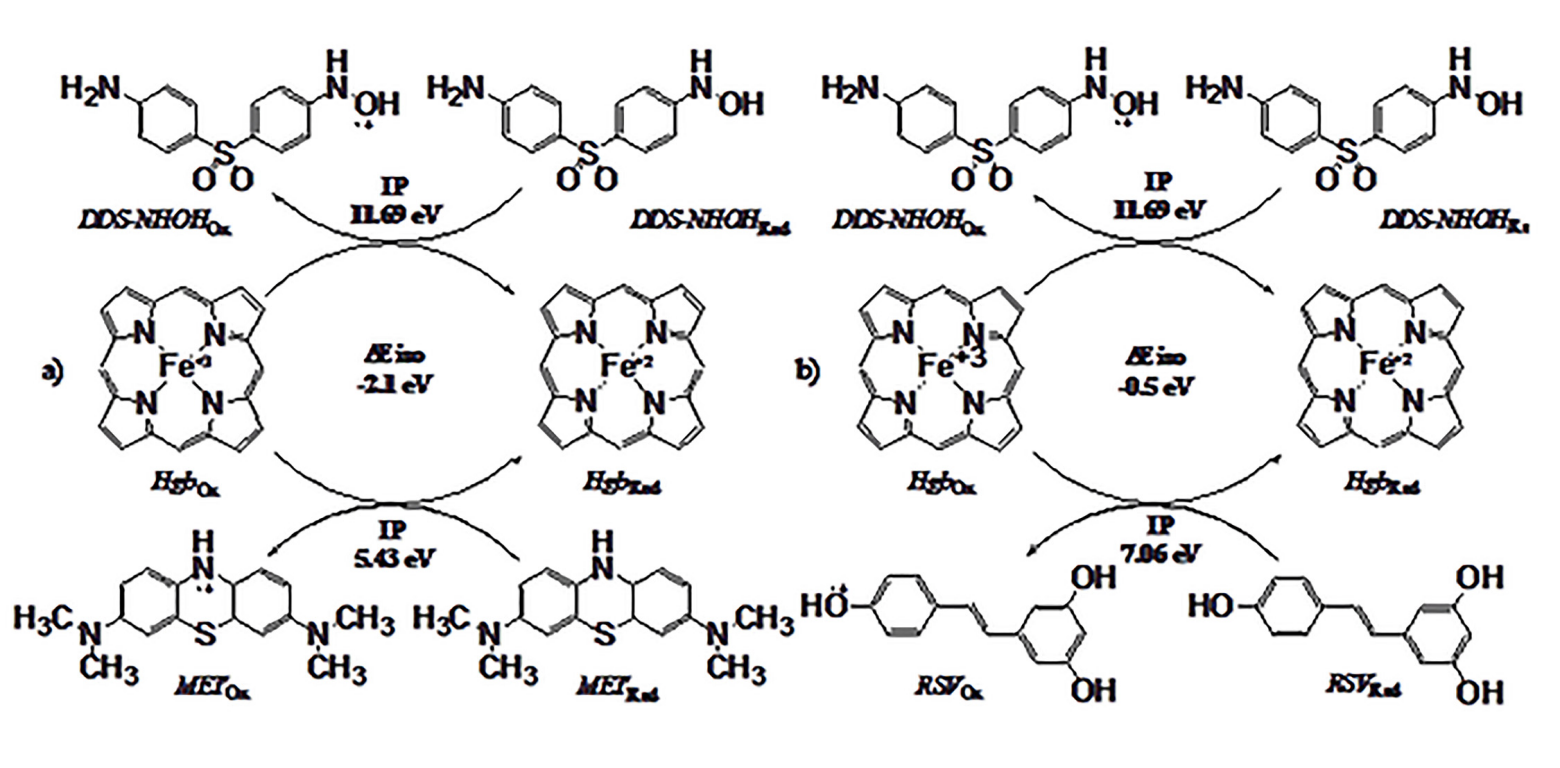
Redox mechanism. Ionization potential and stabilization energy of dapsone hydroxylamine (DDS-NHOH), resveratrol (RSV), and methylene blue (MET) on antioxidant and methemoglobinemia reversion.

Furthermore, the nucleophilicity of MET and RSV can also be expressed by the ionization potential value (IP), which is calculated as the necessary energy for the abstraction of an electron in the molecule. In fact, IP represents the ease of electron donation in these molecules, as electron abstraction is the primary antioxidant mechanism. Thus, our theoretical calculations showed that a high scavenging activity for methemoglobin reversion is associated with smaller IP. Therefore, molecules with low IP values can more easily undergo oxidation. The results showed that MET has an IP value of 5.43 eV, while RSV has an IP value of 7.06 eV, while the lowest scavenging activity or pro-oxidant capacity is related with higher IP values, such as 11.69 eV to DDS-NHOH (see Table 1). These results are related to scavenging capacity of free radicals. However, a different result can be observed for stabilization energy (ΔEiso) which relates the energy values for the chemical reactivity through the one-electron redox between two different molecules, in this case, DDS-NHOH and MET or RSV. In fact, a low scavenging capacity is observed for RSV when compared to MET. The ΔEiso value for RSV (-0.5 eV) is four-fold lower than MET (-2.1 eV). Therefore, these observations explain the difference in methemoglobinemia reversion capacity between MET and RSV (Fig 9).

## Discussion

In this study, DDS-NHOH was able to both induce methemoglobin formation in erythrocytes and DNA damage in lymphocytes, most likely through elevated intracellular ROS production. In addition, our data also showed that the pretreatment with the RSV protected erythrocytes and lymphocytes from methemoglobin formation and DNA damage, respectively, induced by DDS-NHOH. However, MET was much more effective in the reduction of hydroxylamine-induced methemoglobin formation in erythrocytes.

Regarding the pharmacokinetics of DDS, this drug can cross both blood-brain and placental barriers and 70% of the drug is plasma protein-bound [40,41]. DDS is extensively metabolized, and its hydroxylated metabolites are found in plasma at concentrations ranging from 0.4–1.2 mg/L 24 hours after the ingestion of 100 mg of the drug [6,42]. DDS-NHOH and other hydroxylated metabolites are potent oxidants cause the hematologic adverse effects associated with DDS, including methemoglobinemia and hemolytic anemia [6,7,41,43]. DDS-NHOH has long been considered to be the responsible for inducing methemoglobin in patients using DDS [44,45] besides accelerating erythrocytic destruction in rats [19,46] and morphological alteration in human erythrocytes [19,47]. Walker et al. [48] also reported the methemoglobin formation induced by 100 mg/day DDS which resulted in up to 16% levels of methemoglobin in some patients, yielding symptoms such as tachycardia, dyspnea and back pain, as well as anemia and tissue hypoxia. This latter effect is a consequence of methemoglobin-induced compromised O_2_ transport to tissues, as well as the methemoglobin may be a biomarker of anemia-induced tissue hypoxia [49,50].

Hemoglobin is normally involved in a series of oxidation-reduction reactions leading to minor levels of oxidative stress in erythrocytes, producing reactive species and methemoglobin [51], which is reversed through NADH diaphorase [52]. The blood toxicity induced by DDS-NHOH appears be related to the ability of this metabolite to generate high amounts of ROS in erythrocytes, which leads to the appearance of hematologic adverse reactions [53]. Here, our data showed that the methemoglobin formation and ROS production induced by DDS-NHOH was directly proportional, and these effects were dose dependent. These findings may suggest that ROS could be intermediates in the various toxic processes caused by this metabolite, such as methemoglobin formation, DNA damage induction, as well as protective antioxidant enzyme inhibition. Indeed, this latter process increases the vulnerability of cellular macromolecules to ROS formed by DDS-NHOH.

In this regard, Gandhi and Singh [11] reported that leprosy patients on MDT possessed more cells with longer DNA migration lengths than cells from untreated patients. Moreover, some studies reported structural chromosomal abnormalities in cultured skin fibroblasts from leprosy patients treated with DDS [54] and in human lymphocytes *in vitro* [10,55]. Toxic radicals such as superoxide anion, hydrogen peroxide, singlet oxygen and hydroxyl radicals generated in association with drugs such as DDS or its metabolites, may also cause DNA damage [11,56], causing strand breaks and/or alkali-labile lesions [57]. The Comet assay has been established as a simple, rapid, cheap, flexible and, most importantly, sensitive method to detect DNA damage [58] and it is considered to be a highly effective short term mutagenicity assay [59]. In this report, we also demonstrated through this assay that the DDS-NHOH was able to induce DNA damage in peripheral blood lymphocytes.

Regarding the RSV, this antioxidant (10 to 1000 μM) was able to protect *in vitro* human erythrocytes and lymphocytes of the oxidative stress induced by DDS-NHOH, thus, preventing methemoglobin formation and DNA damage, respectively. On the other hand, the RSV was unable to reverse the methemoglobin formation induced by DDS-NHOH. Our results are in broad agreement with Pandey and Rizvi [60], who showed the protective effect of RSV (10 and 100 μM) in human erythrocytes against oxidative stress, preventing the formation of protein carbonyl groups as well as the lipid peroxidation on the erythrocytes membrane. In addition, Qadri et al. [61] also showed that RSV can inhibit erythrocytes suicidal death (eryptosis) by reducing exposure of phosphatidylserine on the cell surface, as well as reversing the rise in Ca^+2^ cytosolic levels seen during eryptosis. RSV can stabilize hemoglobin through the connection of the three hydroxyls groups of stilbene chains α of Pro95, Thr134 and Asp126 forming hydrogen bonds [62], leading to attenuation of high levels of oxidation. Other reports showed that other antioxidants, such as vitamin supplements (vitamins C and E) may also prove protective against methemoglobinemia and genotoxicity caused by anti-leprotic drugs in leprosy patients [11,63,64].

RSV occurs as cis and trans-RSV, but trans-RSV is more biologically active than its cis form. In this regards, pharmacokinetic studies have shown that the trans-RSV has high oral absorption but low bioavailability, on account of rapid and extensive metabolism to RSV-4′-O-glucuronide, RSV-3′-O-glucuronide, and RSV-3-O-sulfate in liver and intestinal epithelial cells in man following single or multiple oral doses (0.5–5g) of RSV [65–67]. Some studies related that three servings of red wine (approx 450 ml) are more than sufficient to achieve plasma levels of free trans-RSV within the range of 100 nM–1μM [65 68].

Clinic studies reported that RSV possess health benefits (both in animal and human studies) at modest doses, whilst higher doses were capable of exerting a pro-apoptotic tumoridal effect [69,70]. In this regard, Nakagawa et al. [71] reported that at low concentrations, RSV significantly increased cell proliferation in human breast cancer cell lines (≤ 22 μM), whereas it suppressed cell growth at high concentrations (≥ 44 μM).

In recent years, various mechanisms have been suggested to explain the antioxidant activity of most of the polyphenols, like RSV, which is associated with at least three processes: 1) increased level of intracellular GSH, 2) attenuation of Ca^2 +^ influx, 3) removal of ROS or inactivation of free radicals by the donation of hydrogen atoms [60,72]. Regarding the RSV, some targets have been discovered *in vitro and in vivo*, including cyclooxygenase-1 (COX1), cyclooxygenase-2 (COX2), the transcription factor NF-κB and Sirtuins (SirTs), mainly SirT 1; these are (NAD)-dependent histone deacetylases that can increase the cell longevity, [72–74]. Targets of highest affinity for RSV also have been described, including quinone reductase 2 (QR2), a FAD-dependent cytosolic enzyme that catalyzes the 1-, 2-, or 4-electron reduction of quinones and other compounds using N-alkyl- and N-ribosylnicotinamides [75,76].

Regarding the DNA-protection mechanism for RSV induced by an oxidative compound such as DDS-NHOH, other authors also reported that NaAsO_2_ exposure led to a decrease in cell proliferation and increase in DNA/chromosomal damage and apoptotic cell death, mainly via oxidative stress. Whereas cells treated with RSV showed improved cell survival and reduced DNA/chromosomal damage, oxidative stress and apoptosis [76]. This protective mechanism of RSV may be associated the SirT1 activation, modulating gene silencing, DNA damage repair, chromosomal stability and several other metabolic processes [74, 77–79]. Studies showed that RSV could extend life span of *Drosophila C*. *elegans* as well as vertebrates such fish and mammals [69,79,80]-. Mukherjee et al. [81] showed that resveratrol also induced the activation of SirT1, SirT3, and SirT4, and the phosphorylation of FoxO1 and fork head box protein O3a (FoxO3a), as well as an anti-aging enzyme pre B cell-enhancing factor (PBEF). Thus, some positive effects of resveratrol in activation of SirT1 protein included age related disorders, as type 2 diabetes, cardiovascular disease, neurodegeneration, and inflammation [80].

In the present study, MET was able to reverse as well as inhibit methemoglobin formation induced by DDS-NHOH at least 2,000 times lower concentration than RSV. MET is a heterocyclic aromatic thiazine water-soluble dye; when it is oxidized, it exhibits a deep blue color although it is colorless in its reduced state (leukomethylene blue, leucoMET). Oxidized MET and leucoMET together form a reversible oxidation–reduction system or electron donor–acceptor couple [82]. In clinics, the redox properties of MET have been utilized in treatments of methemoglobinemia caused by genetic deficiencies and metabolic poisoning and ifosfamide-induced encephalopathy [82–83]. In humans, mean plasma concentration of MET is 5 μM by intravenous bolus injection of 1.4 mg/kg MET [82]. There are various mechanisms whereby MET can act as an electron donor in the nonenzymatic reduction of methemoglobin, as well as exhibiting some antioxidant effects. It has been shown that MET inhibits superoxide production by serving as an artificial electron acceptor by diverting electron flow away from the enzyme sites of various oxidases where molecular oxygen is converted to superoxide radicals [83–86]. In addition, MET absorbs energy directly from a light source and then transfers this energy to molecular oxygen, creating singlet oxygen (^1^O_2_). This oxygen variant is extremely electrophilic and can oxidize directly electron rich double bonds in biological molecules and macromolecules [82,85,86]. Thus, this generation of ROS by MET is one of the main mechanisms of toxicity of this dye. MET at concentrations above 5 μM increased intracellular ROS and OS as evidenced by oxidation of glutathione (GSH), vitamin C and dihydrofluorescein. Thus, ROS is involved in many bioactions, such as phagocytosis, mutagenesis and genotoxicity [87]. Singlet oxygen can cause DNA cleavage and base injury, which may play a role in aging and cancer, and the exposure to ultraviolet radiation and MET may lead the formation of this radical. These processes may play a role in carcinogenesis and others diseases [87, 88]. In addition, MET has been a potential treatment of some neurodegenerative disorders, due the inhibition of aggregation of proteins, including tau protein. However, serious adverse effects of MET has been reported in these patients due to its serotonin toxicity attributed to the inhibition of monoamine oxidase (MAO) [83,89,90].

In this study, the involvement of antioxidant enzyme systems such as SOD and CAT was explored in the detoxification of ROS in erythrocytes, since SOD catalyzes the dismutation reaction of the anion superoxide forming H_2_O_2,_ which is then degraded by CAT forming H_2_O and O_2_ molecules, thereby preventing the formation of hydroxyl radicals and prevent lipid peroxidation [91]. In this regard, our data showed the oxidative action of DDS-NHOH on erythrocytes, which it was able inhibit the CAT activity, but not SOD activity. Recently, we showed that the treatment with MDT in leprosy patients also led to a significant decrease in CAT activity in leprosy patients, but did not alter the SOD activity compared to untreated patients. In addition, we also showed alongside an increase in GSH, in MetHb levels and Heinz body formations [6]. This inhibition of CAT activity may be related to an increase of H_2_O_2_ production, as reported by Bukowska et al. [92], who showed that some oxidative compounds induce methemoglobinemia, leading to increased CAT activity in erythrocytes. In this regard, different drugs, including non-steroidal anti-inflammatory, antineoplastic, antipsychotics, antiretroviral agents, analgesics and antibiotics can enhance the process of ROS production, and they are responsible for oxidative stress-mediated toxicity in various tissues and organs including heart, kidney, liver and brain [93]. Furthermore, some studies reported that the reduction of CAT activity might be associated with factors related to individuals, such as enzymatic deficiencies due to genetic mutations or a reduced synthesis of this enzyme by changes in their gene expression [94]. Many factors have been reported that can affect the gene expression of CAT, such as the presence of certain ions, cytokines and drugs [95]. These findings suggest that oxidative stress induced by DDS-NHOH, reducing enzymatic antioxidants factors and increasing free radical, may be associated to interference, activation or inhibition of non-enzymatic and enzymatic synthesis, or expression of target genes.

Here, we also demonstrated a lack of RSV action on CAT and SOD activity in our model, suggesting that the mechanism of inhibition of ROS and methemoglobin by RSV is unlikely to be mediated by increased activity of SOD and CAT in erythrocytes. *In vitro* studies also showed a protective effect of RSV at 1 and 5 μM, reducing apoptosis and levels of oxidative stress factors, while cells exposed to high concentrations of RSV exhibited increased cell apoptotic and ROS level accompanied with reduced SOD activity and GSH content [43]. RSV can reduce the production of prostaglandin E2 (PGE2) and ROS formation in lipopolysaccharide (LPS)-activated microglial cells. It also suppressed the activity of T and B-cells, and macrophages [65–72]. On this other hand, some studies also showed that resveratrol provides cardioprotection via up regulation of catalase activity in the myocardium [73–74]. RSV was able to promote multiple antioxidant enzymes, including the SOD isoforms, GPx1, catalase, heme oxygenase-1 (HO-1), NAD(P)H: quinone oxidoreductase, and γ-glutamylcysteine synthetase [72–74, 96].

Our *in silico* data supported the biological results, suggesting that for these compounds the electron transfer is the preferred mechanism for the radical-scavenging process, involving electron donating groups. Thus, DDS-NHOH shows a significant contribution regarding the HOMO orbital with respect to the phenyl-hydroxylamine than the aniline ring, whilst MET has a symmetric contribution on both dimethylamine moieties and phenothiazine rings, whereas in RSV the *p*-phenol ring has a greater contribution than the resorcinol ring plus ethane moiety [97].

The oxidative efficiency of these compounds depends not only on the HOMO values, but it is mainly concerned with the IP and ΔEiso values. Therefore, the alteration of resonance effects between oxygen, nitrogen and sulphur atoms stabilizes the cation free radical by transfer of additional negative charge. Thus, electron-donating groups have a direct influence in the resonance effect among phenyl-hydroxylamine, dimethylamines, and phenothiazine, aniline *p*-phenol and *m*-phenol rings.

In accordance with these studies, here the structure-activity relationship of scavenging activity for DDS-NHOH, RSV and MET have been compared with their values of HOMO, IP, and ΔEiso for electron transfer using quantum chemical calculations at the DFT/B3LYP theory level with the 6–31+G (d,p) basis sets. The scavenging properties of cation free radicals (M^•+^) are controlled by the oxidation capacity and reduction on Fe^+3^ for Fe^+2^. The lower the IP values and the higher the ΔEiso values are, is related to potent electron-transfer ability. These values can explain the higher scavenging capacity of MET by both mechanisms when compared to RSV.

**Fig 10:**
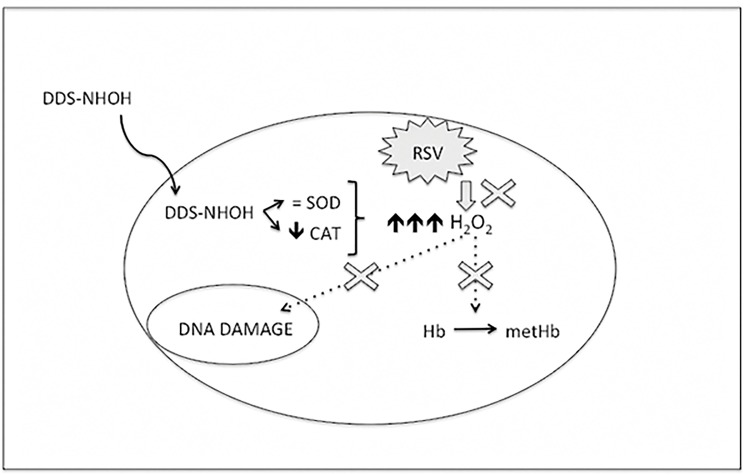
Proposal for a possible action mechanism of resveratrol (RSV) in inhibiting methemoglobin formation and DNA damage induced by DDS hydroxylamine (DDS-NHOH) *in vitro* model.

In summary, the present study has demonstrated some of the protective effects of RSV on DDS-NHOH-mediated toxicity in human erythrocytes and lymphocytes *in vitro*. The Fig 10 shows a hypothesis of proposal for a possible action mechanism of resveratrol (RSV) in inhibiting methemoglobin formation and DNA damage induced by DDS hydroxylamine (DDS-NHOH) *in vitro* model. Whilst other studies have applied agents such as ascorbic acid [53], α-lipoic acid and DHLA [12] to the attenuation of hydroxylamine toxicity. This report demonstrates some of the potential of RSV to reduce the toxicity of DDS in leprosy patients, although further pharmacokinetic, pharmacodynamic and toxicological investigation will be necessary before future clinical recommendations on possible RSV supplementation can be made. Our theoretical mechanistic approach also accounts for the higher scavenging capacity of MET in comparison with that of RSV, which may explain its high effectiveness in the inhibition and reversal of the methemoglobin formation.

## Supporting Information

S1 Table Data of MetHb formation induced by DDS-NHOH. Erythrocytes were incubated with different concentrations of DDS-NHOH (2.5; 5.0 and 7.5 μg/mL) for 1 h at 37°C.
(DOCX)

S2 Table Data of the pretreatment with different concentration of resveratrol (RSV) on methemoglobin formation induced by DDS-NHOH. Erythrocytes were pretreated with different concentrations of RSV(10, 100, 200 and 1000 μM) for 1 h at 37°C, then these cells were incubated with different concentrations of DDS-NHOH (2.5; 5.0 and 7.5 μg/mL) for 1 h at 37°C.
(DOCX)

S3 Table Data of the comparative effect of the pretreatment with resveratrol (RSV) or methylene blue (MET) on methemoglobin formation induced by DDS-NHOH. Erythrocytes were pre-incubated with RSV (100 μM) for 1 h or MET (40 nM) for 30 min, after these cells were incubated for 1 h with different concentrations of DDS-NHOH (2.5, 5.0 and 7,5 μg/mL).
(DOCX)

S4 Table Data of the comparative effect of post-treatment with resveratrol (RSV) or methylene blue (MET) on methemoglobin formation induced by DDS-NHOH. Erythrocytes were incubated for 1 h with DDS-NHOH (2.5 μg/mL), then these cells were incubated with RSV (100μM) for 1 h or MET(40 nM).
(DOCX)

S5 Table Data of the treatment with resveratrol on DNA damage induced by DDS-NHOH. Tail Length (μm—**A**), DNA in tail (%—**B**) Tail Moment (TM—**C**) and Olive Moment (OM—**D**) were used as a marker of DNA damage in lymphocyte using Comet assay. As positive control was used H_2_O_2_ (200 μM).
(DOCX)

S6 Table Data of the Reactive oxygen species (ROS) generation. Erythrocytes were pretreated with resveratrol (RSV, 100 μM and 1000 μM) for 1 h at 37°C and incubated for 30 min with DDS-NHOH (2.5 μg/ml and 7.5 μg/ml). As positive control was used T-BHP (200 μM). ROS production was measured as dichlorofluorescein (DCF) fluorescence.
(DOCX)

S7 Table Data of CAT and SOD activity. Erythrocytes were pretreated with resveratrol (RSV, 100 μM and 1000 μM) for 1 h at 37°C and incubated for 30 min with DDS-NHOH (2.5 μg/ml) or T-BHP (200 μM).
(DOCX)
