# Supplementary material for: In Vitro Protective Effect and Antioxidant Mechanism of Resveratrol Induced by Dapsone Hydroxylamine in Human Cells
Source: PLoS One. 2015 Aug 18;10(8):e0134768. doi: 10.1371/journal.pone.0134768 (PMC4540410; doi:10.1371/journal.pone.0134768)
Supplement: S1 Table — Erythrocytes were incubated with different concentrations of DDS-NHOH (2.5; 5.0 and 7.5 μg/mL) for 1 h at 37°C. (DOCX) [file pone.0134768.s001.docx]

| **S1 Table** |  |  |  |  |  |  |  |  |  |  |  |  |
| --- | --- | --- | --- | --- | --- | --- | --- | --- | --- | --- | --- | --- |
| DDS-NOH (μg/mL) | ERI+ DDS | | | |  |  |  |  |  |  |  |  |
|  |  |  |  |  |  |  |  |  |  |  | MEAN | SEM |
| 2.5 | 11.4000 | 15.2700 | 21.6900 | 28.6100 | 31.2333 | 29.3100 | 14.8770 | 18.8680 | 17.9800 |  | 21.026 | 2.381 |
| 5 | 26.0400 | 25.0000 | 25.6700 | 24.6770 | 25.8700 | 26.1000 | 25.1000 | 25.1700 | 25.0100 |  | 25.404 | 0.174 |
| 7.5 | 34.6400 | 32.1600 | 34.6800 | 32.8600 | 34.6800 | 32.8600 | 32.8750 | 33.8220 | 33.8890 |  | 33.607 | 0.317 |
| 10 | 43.97 | 37.98 | 44.01 | 43.08 | 44.001 | 39.121 | 37.19 | 43.11 | 37.011 |  | 41.053 | 1.046 |

***MS:* “*In vitro* protective effect and antioxidant mechanism of resveratrol on oxidative stress generation induced by Dapsone hydroxylamine in human blood cells”** *by Rosyana V. Albuquerque, Nívea Silva Malcher, Lílian Lund Amado, Michael D. Coleman, Danielle Cardoso dos Santos, Rosivaldo dos Santos Borges, Sebastião Aldo da Silva Valente, Vera da Costa Valente, Marta Chagas Monteiro*

**S1 Table. Data of MetHb formation induced by DDS-NHOH.** Erythrocytes were incubated with different concentrations of DDS-NHOH (2.5; 5.0 and 7.5 µg/mL) for 1 h at 37 °C.
