# Supplementary material for: In Vitro Protective Effect and Antioxidant Mechanism of Resveratrol Induced by Dapsone Hydroxylamine in Human Cells
Source: PLoS One. 2015 Aug 18;10(8):e0134768. doi: 10.1371/journal.pone.0134768 (PMC4540410; doi:10.1371/journal.pone.0134768)
Supplement: S2 Table — Erythrocytes were pretreated with different concentrations of RSV(10, 100, 200 and 1000 μM) for 1 h at 37°C, then these cells were incubated with different concentrations of DDS-NHOH (2.5; 5.0 and 7.5 μg/mL) for 1 h at 37°C. (DOCX) [file pone.0134768.s002.docx]

***MS:* “*In vitro* protective effect and antioxidant mechanism of resveratrol on oxidative stress generation induced by Dapsone hydroxylamine in human blood cells”** *by Rosyana V. Albuquerque, Nívea Silva Malcher, Lílian Lund Amado, Michael D. Coleman, Danielle Cardoso dos Santos, Rosivaldo dos Santos Borges, Sebastião Aldo da Silva Valente, Vera da Costa Valente, Marta Chagas Monteiro*

| **S2 Table** |  | | | | | |  |  |  |  | MEAN | SEM |
| --- | --- | --- | --- | --- | --- | --- | --- | --- | --- | --- | --- | --- |
| ERi+methanol | 5.0350 | 5.1282 | 7.6892 | 8.8034 | 5.1282 | 8.8140 | 5.4500 | 8.9600 | 6.3200 |  | 6.814 | 0.581 |
| Eri + RSV 10 | 1.3002 | 1.6807 | 2.3907 | 2.9900 | 2.0010 | 2.5550 | 1.3430 | 0.9760 | 0.9880 |  | 1.803 | 0.241 |
| DDS (2,5) | 18.6100 | 18.9800 | 31.23 | 21.6900 | 35.2700 | 18.4000 | 18.8680 | 16.8770 | 19.3100 |  | 22.138 | 2.167 |
| DDS(2,5)+RSV 10 | 10.9567 | 10.1852 | 10.4000 | 10.1000 | 11.8520 | 10.2020 | 10.1780 | 10.7600 | 10.1220 |  | 10.528 | 0.194 |
| DDS(2,5)+RSV 100 | 5.8193 | 6.4964 | 5.9930 | 7.4964 | 9.8800 | 8.9000 | 7.2592 | 7.3890 | 5.5660 |  | 7.1999 | 0.482 |
| DDS(2,5)+RSV 200 | 10.7000 | 10.0000 | 10.0000 | 10.4000 | 10.1000 | 10.2110 | 9.8990 | 11.1980 | 10.8990 |  | 10.379 | 0.152 |
| DDS(2,5) + RSV 1000 | 7.4799 | 8.4308 | 9.2025 | 8.9990 | 8.4600 | 9.1660 | 7.9720 | 7.5600 | 8.2400 |  | 8.390 | 0.216 |
| DDS (5,0) | 25.6700 | 26.3000 | 25.8700 | 24.7700 | 25.1000 | 26.8900 | 25.1700 | 25.3400 | 25.4510 |  | 25.618 | 0.174 |
| DDS(5,0)+ RSV 10 | 24.8350 | 22.1712 | 22.8114 | 24.1030 | 23.7200 | 23.2210 | 21.5540 | 22.9990 | 23.0010 |  | 23.157 | 0.328 |
| DDS(5,0)+ RSV 100 | 18.3429 | 18.5238 | 18.8448 | 18.6660 | 18.6000 | 18.1450 | 17.9540 | 18.9010 | 19.0080 |  | 18.554 | 0.118 |
| DDS(5,0)+ RSV 200 | 21.8391 | 21.1500 | 20.4096 | 21.0890 | 19.6230 | 20.4200 | 18.6156 | 19.9110 | 19.0920 |  | 20.239 | 0.346 |
| DDS(5,0)+ RSV 1000 | 20.0321 | 20.1258 | 19.9575 | 20.1650 | 20.0450 | 20.1700 | 19.5750 | 20.1220 | 20.0010 |  | 20.021 | 0.061 |
| DDS (7,5) | 33.6800 | 34.8600 | 30.8220 | 33.8990 | 34.8750 | 32.8600 | 33.1600 | 34.6800 | 34.6400 |  | 33.720 | 0.439 |
| DDS(7,5)+ RSV 10 | 27.8274 | 31.1438 | 27.1552 | 29.1210 | 28.8770 | 29.7640 | 26.1210 | 28.9880 | 28.8760 |  | 28.652 | 0.489 |
| DDS(7,5)+ RSV 100 | 24.5781 | 23.8994 | 22.3684 | 22.5820 | 22.7390 | 23.1010 | 24.3760 | 24.8780 | 23.7940 |  | 23.590 | 0.309 |
| DDS(7,5)+ RSV 200 | 27.2500 | 26.3888 | 26.1230 | 27.1110 | 24.6710 | 24.9323 | 26.9880 | 26.9880 | 25.9230 |  | 26.264 | 0.316 |
| DDS(7,5)+ RSV 1000 | 23.4705 | 19.4134 | 23.3740 | 22.1705 | 23.0010 | 20.1210 | 23.1980 | 20.1890 | 24.0010 |  | 22.104 | 0.576 |

**S2 Table. Data of the pretreatment with different concentration of resveratrol (RSV) on methemoglobin formation induced by DDS-NHOH*.*** Erythrocytes were pretreated with different concentrations of RSV(10, 100, 200 and 1000 µM) for 1 h at 37 °C, then these cells were incubated with different concentrations of DDS-NHOH (2.5; 5.0 and 7.5 µg/mL) for 1 h at 37 °C.
