# Supplementary material for: In Vitro Protective Effect and Antioxidant Mechanism of Resveratrol Induced by Dapsone Hydroxylamine in Human Cells
Source: PLoS One. 2015 Aug 18;10(8):e0134768. doi: 10.1371/journal.pone.0134768 (PMC4540410; doi:10.1371/journal.pone.0134768)
Supplement: S3 Table — Erythrocytes were pre-incubated with RSV (100 μM) for 1 h or MET (40 nM) for 30 min, after these cells were incubated for 1 h with different concentrations of DDS-NHOH (2.5, 5.0 and 7,5 μg/mL). (DOCX) [file pone.0134768.s003.docx]

***MS:* “*In vitro* protective effect and antioxidant mechanism of resveratrol on oxidative stress generation induced by Dapsone hydroxylamine in human blood cells”** *by Rosyana V. Albuquerque, Nívea Silva Malcher, Lílian Lund Amado, Michael D. Coleman, Danielle Cardoso dos Santos, Rosivaldo dos Santos Borges, Sebastião Aldo da Silva Valente, Vera da Costa Valente, Marta Chagas Monteiro*

| **S3 Table** |  |  |  |  |  |  |  |  |  |  | **MEAN** | **SEM** |
| --- | --- | --- | --- | --- | --- | --- | --- | --- | --- | --- | --- | --- |
| DDS (2,5) | 18.6100 | 18.9800 | 31.23 | 21.6900 | 35.2700 | 18.4000 | 18.8680 | 16.8770 | 19.3100 |  | 22.138 | 2.167 |
| DDS (5,0) | 25.6700 | 26.3000 | 25.8700 | 24.7700 | 25.1000 | 26.8900 | 25.1700 | 25.3400 | 25.4510 |  | 25.618 | 0.174 |
| DDS (7,5) | 33.6800 | 34.8600 | 30.8220 | 33.8990 | 34.8750 | 32.8600 | 33.1600 | 34.6800 | 34.6400 |  | 33.720 | 0.439 |
| DDS(2,5)+RSV 100 | 5.8193 | 6.4964 | 5.9930 | 7.4964 | 9.8800 | 8.9000 | 7.2592 | 7.3890 | 5.5660 |  | 7.1999 | 0.482 |
| DDS(5,0)+ RSV 100 | 18.3429 | 18.5238 | 18.8448 | 18.6660 | 18.6000 | 18.1450 | 17.9540 | 18.9010 | 19.0080 |  | 18.554 | 0.118 |
| DDS(7,5)+ RSV 100 | 24.5781 | 23.8994 | 22.3684 | 22.5820 | 22.7390 | 23.1010 | 24.3760 | 24.8780 | 23.7940 |  | 23.591 | 0.309 |
| DDS(2,5)+MB 40nM | 6.1261 | 6.13367 | 6.13367 | 6.011 | 6.321 | 6.13 | 6.161 | 6.134 | 6.099 |  | 6.139 | 0.027 |
| DDS(5,0)+ MB 40nM | 15.72649 | 15.84249 | 16.5307 | 16.119 | 16.64 | 16.172 | 15.796 | 15.721 | 15.791 |  | 16.038 | 0.117 |
| DDS(7,5)+ MB 40nM | 17.8839 | 17.8506 | 17.8378 | 17.111 | 17.665 | 17.998 | 18.007 | 17.966 | 17.988 |  | 17.812 | 0.095 |

**S3 Table. Data of the comparative effect of the pretreatment with resveratrol (RSV) or methylene blue (MET) on methemoglobin formation induced by DDS-NHOH.** Erythrocytes were pre-incubated with RSV (100 µM) for 1 h or MET (40 nM) for 30 min, after these cells were incubated for 1 h with different concentrations of DDS-NHOH (2.5, 5.0 and 7,5 µg/mL).
