# Supplementary material for: In Vitro Protective Effect and Antioxidant Mechanism of Resveratrol Induced by Dapsone Hydroxylamine in Human Cells
Source: PLoS One. 2015 Aug 18;10(8):e0134768. doi: 10.1371/journal.pone.0134768 (PMC4540410; doi:10.1371/journal.pone.0134768)
Supplement: S4 Table — Erythrocytes were incubated for 1 h with DDS-NHOH (2.5 μg/mL), then these cells were incubated with RSV (100μM) for 1 h or MET(40 nM). (DOCX) [file pone.0134768.s004.docx]

***MS:* “*In vitro* protective effect and antioxidant mechanism of resveratrol on oxidative stress generation induced by Dapsone hydroxylamine in human blood cells”** *by Rosyana V. Albuquerque, Nívea Silva Malcher, Lílian Lund Amado, Michael D. Coleman, Danielle Cardoso dos Santos, Rosivaldo dos Santos Borges, Sebastião Aldo da Silva Valente, Vera da Costa Valente, Marta Chagas Monteiro*

| **S 4 Table** |  |  |  |  |  |  |  |  |  |  | MEAN | SEM |
| --- | --- | --- | --- | --- | --- | --- | --- | --- | --- | --- | --- | --- |
| METHANOL | 1.54 | 1.71 | 1.81 | 1.85 | 2.4 | 1.81 | 1.85 | 2.6 | 1.99 |  | 1.951 | 0.112 |
| DDS 2,5 | 11.4 | 14.27 | 22.69 | 28.61 | 10.3 | 24.5 | 15.9 | 21.42 | 16.99 |  | 18.453 | 2.071 |
| DDS 2,5+RSV 100 | 30 | 33 | 2.1 | 6.1 | 30.7 | 11.67 | 31.3 | 10.8 | 6.1 |  | 17.974 | 4.306 |
| DDS 2,5+MB 40 | 0.01 | 0.001 | 0.5 | 0.5 | 0.6 | 1 | 0.8 | 0.65 | 0.889 |  | 0.55 | 0.117 |

**S4 Table. Data of the comparative effect of post-treatment with resveratrol (RSV) or methylene blue (MET) on methemoglobin formation induced by DDS-NHOH.** Erythrocytes were incubated for 1 h with DDS-NHOH (2.5 µg/mL), then these cells were incubated with RSV (100µM) for 1 h or MET(40 nM).
