# Supplementary material for: In Vitro Protective Effect and Antioxidant Mechanism of Resveratrol Induced by Dapsone Hydroxylamine in Human Cells
Source: PLoS One. 2015 Aug 18;10(8):e0134768. doi: 10.1371/journal.pone.0134768 (PMC4540410; doi:10.1371/journal.pone.0134768)
Supplement: S5 Table — Tail Length (μm—A), DNA in tail (%—B) Tail Moment (TM—C) and Olive Moment (OM—D) were used as a marker of DNA damage in lymphocyte using Comet assay. As positive control was used H2O2 (200 μM). (DOCX) [file pone.0134768.s005.docx]

***MS:* “*In vitro* protective effect and antioxidant mechanism of resveratrol on oxidative stress generation induced by Dapsone hydroxylamine in human blood cells”** *by Rosyana V. Albuquerque, Nívea Silva Malcher, Lílian Lund Amado, Michael D. Coleman, Danielle Cardoso dos Santos, Rosivaldo dos Santos Borges, Sebastião Aldo da Silva Valente, Vera da Costa Valente, Marta Chagas Monteiro*

| **S5 Table** - COMET ASSAY | | |  |  |  |
| --- | --- | --- | --- | --- | --- |
|  |  | **TL** | **%T** | **TM** | **OM** |
| **RPMI** | MEAN | 37.79 | 9.73 | 4.86 | 6.89 |
|  | SEM | 21.01 | 2.22 | 2.24 | 2.24 |
| **H2O2** | MEAN | 143.23 | 46.38 | 80.47 | 56.26 |
|  | SEM | 42.00 | 11.80 | 28.32 | 21.37 |
| **RSV** | MEAN | 43.39 | 9.21 | 5.11 | 6.62 |
|  | SEM | 21.72 | 2.25 | 2.25 | 2.25 |
| **MET** | MEAN | 36.16 | 8.07 | 4.05 | 6.27 |
|  | SEM | 22.32 | 4.63 | 2.63 | 2.63 |
| **DDS** | MEAN | 128.71 | 25.03 | 46.72 | 37.31 |
|  | SEM | 40.0 | 8.08 | 10.25 | 10.25 |
| **DDS+RSV** | MEAN | 46.29 | 11.22 | 7.52 | 9.15 |
|  | SEM | 26.15 | 4.84 | 4.84 | 4.0 |
|  |  |  |  |  |  |

**S5 Table. Data of the treatment with resveratrol on DNA damage induced by DDS-NHOH**. Tail Length (µm - **A**), DNA in tail (% - **B**) Tail Moment (TM - **C**) and Olive Moment (OM - **D**) were used as a marker of DNA damage in lymphocyte using Comet assay. As positive control was used H_2_O_2_ (200 µM). **GENERAL MEAN: 2 BLADES AND 50 CELLS FOR BLADE**
