# Supplementary material for: In Vitro Protective Effect and Antioxidant Mechanism of Resveratrol Induced by Dapsone Hydroxylamine in Human Cells
Source: PLoS One. 2015 Aug 18;10(8):e0134768. doi: 10.1371/journal.pone.0134768 (PMC4540410; doi:10.1371/journal.pone.0134768)
Supplement: S6 Table — Erythrocytes were pretreated with resveratrol (RSV, 100 μM and 1000 μM) for 1 h at 37°C and incubated for 30 min with DDS-NHOH (2.5 μg/ml and 7.5 μg/ml). As positive control was used T-BHP (200 μM). ROS production was measured as dichlorofluorescein (DCF) fluorescence. (DOCX) [file pone.0134768.s006.docx]

***MS:* “*In vitro* protective effect and antioxidant mechanism of resveratrol on oxidative stress generation induced by Dapsone hydroxylamine in human blood cells”** *by Rosyana V. Albuquerque, Nívea Silva Malcher, Lílian Lund Amado, Michael D. Coleman, Danielle Cardoso dos Santos, Rosivaldo dos Santos Borges, Sebastião Aldo da Silva Valente, Vera da Costa Valente, Marta Chagas Monteiro*

| **S6 Table ROS** |  |  |  |  |  |  |  |  |  |  | MEAN | SEM |
| --- | --- | --- | --- | --- | --- | --- | --- | --- | --- | --- | --- | --- |
| METHANOL | 5 | 4.5 | 1.9 | 0.9 | 3.5 | 5.7 | 3.7 | 4.76 | 1.9 |  | 3.54 | 0.548 |
| TBHP | 72.5 | 75.8 | 72.1 | 84.2 | 59.6 | 75.3 | 82.1 | 59.6 | 78.4 |  | 73.289 | 2.911 |
| RSV 100 | 4.2 | 4.8 | 4.1 | 5 | 3.8 | 4.8 | 5.2 | 2.9 | 5.5 |  | 4.478 | 0.269 |
| RSV 1000 | 2.4 | 1.7 | 1.9 | 0.9 | 3.5 | 1.8 | 3.5 | 1.8 | 1.3 |  | 2.089 | 0.30 |
| DDS 2,5 | 20.4 | 27.4 | 19.2 | 21.7 | 17.4 | 24.3 | 30.1 | 15.7 | 18.1 |  | 21.589 | 1.603 |
| RSV 100 +DDS 2,5 | 0.8 | 0.6 | 1.9 | 1 | 0.7 | 1.1 | 1.12 | 0.9 | 0.97 |  | 1.01 | 0.126 |
| RSV 1000 +DDS 2,5 | 0.2 | 0.1 | 1 | 0.7 | 1 | 1.5 | 0.6 | 1.7 | 0.2 |  | 0.778 | 0.191 |
| DDS 7,5 | 85.1 | 83.7 | 69.7 | 55.5 | 84 | 81.7 | 53.9 | 91 | 82 |  | 76.289 | 4.483 |
| DDS7,5 RSV 100 | 81.2 | 77.1 | 79.6 | 77.8 | 76.6 | 85.1 | 80 | 79 | 75.5 |  | 79.55 | 0.962 |
| DDS 7,5 + RSV 1000 | 69.5 | 43.7 | 67 | 41.9 | 68.8 | 63.5 | 52.1 | 41.3 | 63 |  | 56.756 | 3.994 |

**S6 Table. Data of the Reactive oxygen species (ROS) generation.** Erythrocytes were pretreated with resveratrol (RSV, 100 µM and 1000 µM) for 1 h at 37 °C and incubated for 30 min with DDS-NHOH (2.5 µg/ml and 7.5 µg/ml). As positive control was used T-BHP (200 µM). ROS production was measured as dichlorofluorescein (DCF) fluorescence.
